# A nomogram for predicting sedation-related adverse events in elderly patients undergoing painless gastrointestinal endoscopy

**DOI:** 10.3389/fmed.2025.1713720

**Published:** 2026-01-12

**Authors:** Liu Xu, Qiyuan Yin, Hui Liu, Qian Liu, Hongyan Zhang

**Affiliations:** Department of Anesthesiology, Wenjiang Hospital of Sichuan Provincial People’s Hospital, Chengdu Wenjiang District People’s Hospital, Chengdu, China

**Keywords:** sedation, gastrointestinal endoscopy, elderly patients, hypotension, hypoxemia

## Abstract

**Background:**

Elderly patients undergoing painless gastrointestinal endoscopy are at increased risk for sedation-related adverse events (SRAEs) because of their greater physiological vulnerability and higher likelihood of comorbidities. Risk stratification before endoscopy may improve perioperative safety and individualize sedation and management.

**Objective:**

This study aimed to develop and internally validate a Firth-penalized multivariable logistic regression model and nomogram to predict SRAEs in elderly patients undergoing painless gastrointestinal endoscopy.

**Methods:**

Prospective data from 520 patients at least 60 years old who underwent painless gastrointestinal endoscopy between April 2023 and June 2024 at our medical center were randomly divided into a training set (*n* = 364) and validation set (*n* = 156). SRAEs were defined as intraoperative hypotension or hypoxemia, and independent predictors of SRAEs in the training set were identified through Firth’s penalized multivariable logistic regression. A nomogram to predict risk of SRAEs was developed using R software and tested against the validation set. Its performance was assessed in terms of receiver operating characteristic curves, calibration plots, and decision curve analysis.

**Results:**

In the training set, hypotension and hypoxemia occurred in 39.0 and 33.5% of patients, respectively, the incidence of SRAEs was 45.6%. The independent predictors were older age, history of snoring, frailty, preexisting hypertension, chronic obstructive pulmonary disease, prolonged fasting before the procedure, and higher initial dose of etomidate-propofol. Conversely, regular physical activity was a protective factor. The nomogram built from the training set discriminated between people in the validation set who experienced SRAEs or not with an area under the curve of 0.92 (95% CI, 0.86–0.97), it showed good calibration in the Hosmer–Lemeshow test (*P* = 0.63), and decision curve analysis demonstrated clinical utility across a wide range of threshold probabilities (7–93%).

**Conclusion:**

A predictive model based on readily available clinical variables can accurately estimate SRAE risk in elderly patients undergoing painless gastrointestinal endoscopy. The model may be useful for individualizing sedation and patient management.

**Clinical trial registration:**

https://www.chictr.org.cn/showproj.html?proj=188331, identifier ChiCTR2300069816.

## 1 Introduction

Endoscopy has become a widely used clinical tool for the early detection of gastrointestinal malignancies, and a growing number of patients opt for sedation during the procedure to increase diagnostic accuracy and comfort (1). However, sedation increases the risk of hypotension and hypoxemia, and it can lead in rare cases to life-threatening cardiocerebrovascular events (2, 3). For example, the most frequently used sedative for painless gastrointestinal endoscopy is propofol, which causes hypotension in up to 36% of patients and hypoxemia in up to 21% of patients. The risk of these sedation-related adverse events (SRAEs) is likely to be higher among elderly than younger patients (4), because elderly are more likely to be experiencing respiratory depression, upper airway obstruction, decreased chest wall compliance (2, 3, 5), reduced cardiopulmonary reserve, impaired hepatic and renal function, and multiple chronic comorbidities. Therefore, early identification of elderly patients at high risk of SRAEs during painless gastrointestinal endoscopy is critical for individualizing peri-sedation management and improving patient safety.

Although studies have described models to predict risk of intra-procedural hypotension and other SRAEs (6, 7), the literature on SRAEs has not focused on elderly or taken into account variables from throughout the sedation process. Multivariable prediction models that integrate pre-procedural characteristics, intra-procedural management, and sedation-related factors may help refine risk stratification in this vulnerable population. Therefore, we took comprehensive account of peri-procedural variables and developed a Firth-penalized multivariable logistic regression prediction model and nomogram to estimate the risk of SRAEs in elderly patients undergoing painless gastrointestinal endoscopy, which we validated internally. This model is intended to support clinical decision-making and improve anesthetic care for elderly patients at high risk of sedation-related complications.

## 2 Materials and methods

### 2.1 Patients

This was a prospective, single-center, observational study conducted at Wenjiang District People’s Hospital of Chengdu. The study protocol was approved by the institutional ethics committee (approval EC-Research-2023–003), and the trial was prospectively registered in the Chinese Clinical Trial Registry (registration ChiCTR2300069816, registration date: March 27, 2023). Informed consent was obtained from all participants prior to enrollment.

Data were collected from a consecutive series of patients at least 60 years old who underwent painless gastrointestinal endoscopy between April 2023 and June 2024 at our hospital and who had an American Society of Anesthesiologists (ASA) physical status I–III at baseline. The case report form (CRF) used in this study was specifically developed for this project to collect demographic, clinical, anesthetic, and hemodynamic data of elderly patients undergoing painless gastrointestinal endoscopy. The CRF has not been previously published elsewhere. An English version of the CRF is provided as Supplementary File 1. Patients were excluded if they (1) were known to be allergic or hypersensitive to sedative or anesthetic agents; (2) were suffering from gastric retention or acute upper gastrointestinal bleeding involving excessive gastric content; (3) had a history of epilepsy, psychiatric disorders, hearing impairment, dementia, or other conditions affecting their compliance in the study; (4) experienced hemodynamic shock or instability before endoscopy; (5) had severe hepatic or renal insufficiency before endoscopy; (6) had systolic blood pressure > 160 mmHg or < 90 mmHg or diastolic blood pressure > 100 mmHg or < 60 mmHg before endoscopy; (7) had peripheral oxygen saturation (SpO_2_) < 90% with room air before endoscopy; or (8) received medications other than sedatives, such as norepinephrine spray, during endoscopy.

### 2.2 Minimal sample size

The planned sample size was determined *a priori* based on events-per-variable (EPV) (8, 9) considerations for multivariable logistic regression and the anticipated incidence of SRAEs in elderly patients undergoing painless gastrointestinal endoscopy. Assuming an SRAE incidence of approximately 30% in this high-risk population and a maximum of eight predictors in the final model, and adopting a conservative threshold of 15 events per variable, we required at least 120 SRAE events, corresponding to about 400 analyzable patients. Allowing for an anticipated 20% dropout or exclusion rate, the target enrollment was therefore set at a minimum of 500 elderly patients.

### 2.3 Sedation and management

Before endoscopy, all patients were evaluated at our anesthesia outpatient clinic, during which they provided written informed consent to undergo the procedure and to participate in our study. Patients fasted for at least 8 h and abstained from liquids for at least 4 h before the procedure. Patients scheduled for colonoscopy also received oral sodium phosphate solution and simethicone emulsion for bowel cleansing. In the procedure room, patients were positioned in the left lateral decubitus position, intravenous access was established via the right dorsal hand vein, and lactated Ringer’s solution was infused. Heart rate, SpO_2_ and non-invasive blood pressure on the left upper arm were monitored continuously. Oxygen was administered via nasal cannula at 2–3 L/min. Endoscopic procedures were performed by qualified endoscopists under the supervision of board-certified anesthesiologists, in accordance with standard diagnostic and therapeutic protocols.

Anesthesia was induced using intravenous sufentanil citrate at 0.05 μg/kg, followed by administration of 1% lidocaine at 0.3 mg/kg after 1.5–2 min, which was administered solely to attenuate the injection pain of the subsequent etomidate–propofol mixture, then slow injection of 0.2 mL/kg of an etomidate-propofol mixture that had been prepared by combining 10 mL propofol with 10 mL etomidate. Anesthesia was maintained by administering an additional 0.07 mL/kg of the etomidate-propofol mixture every 5 min. If the patient exhibited body movement or cough reflex during the procedure, additional etomidate-propofol mixture was given as needed at a dose of 0.07 mL/kg.

Intraoperative hypotension was treated with intravenous ephedrine 3–6 mg, while sinus bradycardia, defined as a heart rate below 50 bpm, was treated with intravenous atropine 0.3–0.5 mg. When SpO_2_ fell below 90%, hypoxemia was treated initially with manual chin lifting; if this proved ineffective, endoscopy was paused and oxygen was delivered under positive pressure via facemask until SpO_2_ ≥ 90%. After the procedure, patients were transferred to the post-anesthesia care unit, from which they were discharged in the company of a family member once they met the criteria routinely used at our hospital.

### 2.4 Definition of SRAEs

SRAEs in this study were defined as either hypotension or hypoxemia occurring between the initiation of anesthesia induction and patient discharge from the procedure room. Hypotension was defined as a decrease in systolic blood pressure by at least 20% from the level before anesthesia induction (10). Hypoxemia was defined as SpO_2_ of 75–89% for < 60 s, and severe hypoxemia is defined as SpO_2_ < 75% at any time (11).

### 2.5 Definition of potential risk factors of SRAEs

Potential risk factors of intraoperative SRAEs were extracted from the literature and our clinical experience and integrated into a standardized data collection form. When patients arrived in the waiting area before sedation and endoscopy, trained investigators conducted face-to-face interviews to obtain baseline information about these potential risk factors as well as demographic and clinical characteristics. The collected data included the following: sex, age, body mass index, physical status on the American Society of Anesthesiologists scale, smoking and alcohol history, physical activity level, history of snoring, frailty status, comorbidities, fasting time before the procedure, and type of endoscopic procedure. Smoking meant daily consumption of ≥ 10 cigarettes within 2 weeks before the procedure. Alcohol consumption meant drinking any type of alcohol at least twice per week during the 2 weeks prior to the procedure. Regular physical activity was defined as exercising for at least 30 min at least 3 times per week at moderate intensity (e.g., brisk walking) during the previous month (12). Snoring severity was graded on the following 4-point scale (13): grade 0, no snoring; grade 1, snoring without apnea or related symptoms; grade 2, loud snoring with symptoms (e.g., daytime fatigue) but no apnea; grade 3, snoring with apnea and evidence of end-organ damage. Frailty was assessed using the Fried Frailty Phenotype, and the scores were categorized as follows (14): 0 points, robust; 1–2 points, pre-frail; ≥ 3 points, frail. Prolonged fasting before the procedure was defined as an interval of more than 20 h between the last oral intake and the start of endoscopy. The following perioperative vital signs were recorded before anesthesia and immediately before endoscope insertion: systolic and diastolic blood pressure, mean arterial pressure, heart rate, and SpO_2_. Intraoperative adverse events included hypotension, hypoxemia, patient movement, sinus bradycardia, and repeated coughing, which was defined as ≥ 3 cough episodes during intubation. Patient discomfort immediately after the procedure was also documented.

### 2.6 Development and validation of a nomogram to predict SRAEs

The dataset was randomly divided into a training set (70%) and a validation set (30%) using a computer-generated allocation sequence, and patients were assigned to a group who experienced at least one SRAE or not. Data were compared between the two groups using SPSS 25.0 (IBM, Armonk, NY, United States). Continuous variables with a normal distribution were reported as mean ± standard deviation, and intergroup differences were assessed for significance using the independent-samples *t*-test. Continuous variables with a skewed distribution were reported as median and interquartile range (IQR), and intergroup differences were assessed using the Mann–Whitney U test. Categorical variables were reported as n (%), and differences were assessed using the χ^2^ test. Differences that were associated with two-sided *P* < 0.05 were considered statistically significant.

Variables differing significantly between groups in the training set at the significance level *P* < 0.05 were entered into Firth’s penalized multivariable logistic regression to identify independent risk factors of SRAEs, which were incorporated into a predictive nomogram constructed in R 4.2.0, The model was internally validated in the validation cohort using bootstrap resampling (1,000 iterations). The model’s performance in both training and validation sets was evaluated in terms of the area under the receiver operating characteristic curve (AUC), calibration plots, the Hosmer–Lemeshow goodness-of-fit test, and clinical utility based on decision curve analysis. Where appropriate, results were reported together with their associated 95% confidence interval (CI).

## 3 Results

**FIGURE 1:**
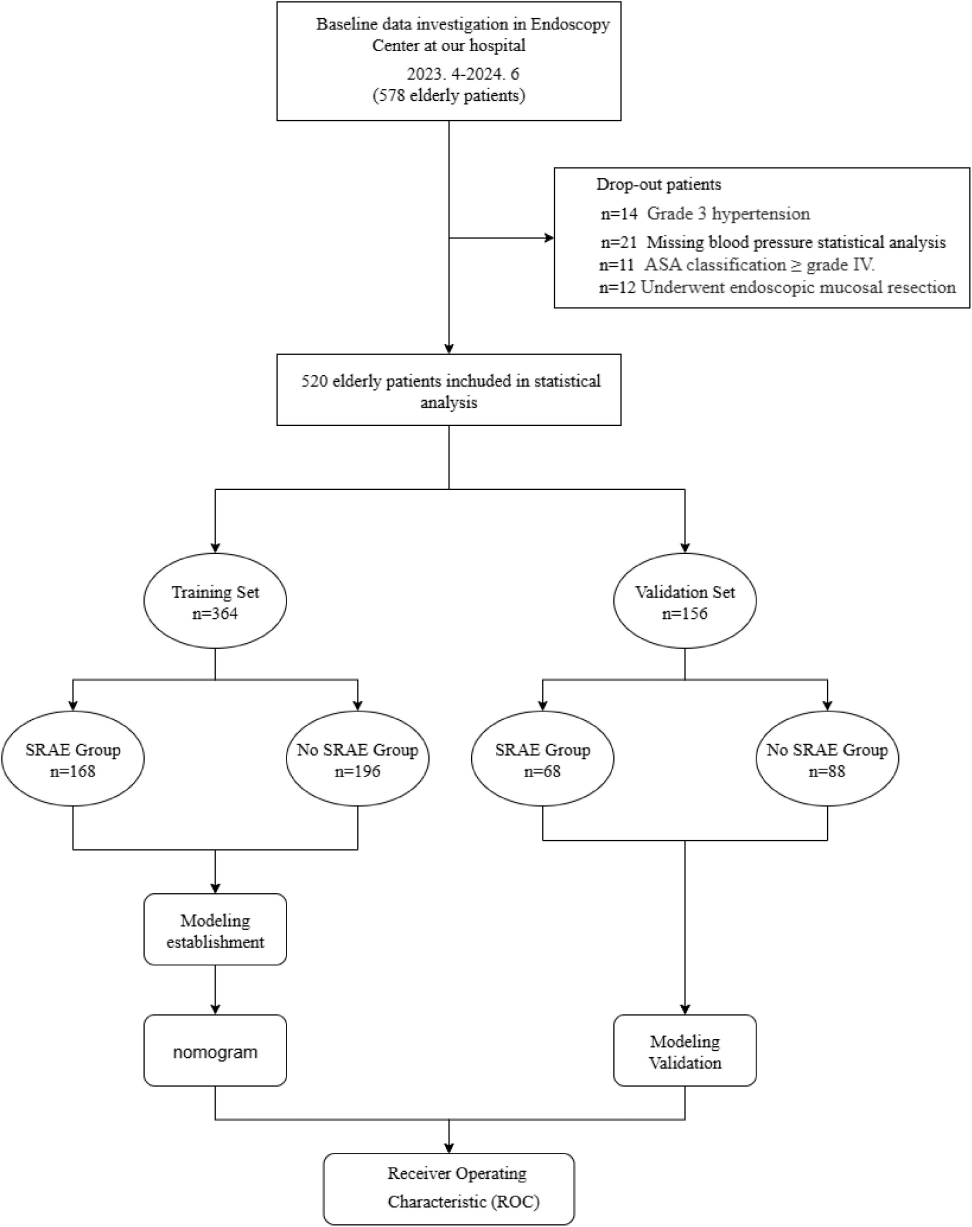
Flowchart of patient enrollment, allocation and analysis. SRAE, sedation-related adverse event.

Of the 578 elderly patients who underwent sedation-assisted gastrointestinal endoscopy at our hospital during the enrollment period, 58 (10.0%) were excluded because their medical data were incomplete or because they were lost to follow-up. The remaining 520, who were 68 ± 6.5 years old (range, 60–93 years) and 266 of whom were men, were included in the final analysis (Figure 1). Just over half the sample underwent both gastroscopy and colonoscopy (286, 55.0%), while a smaller proportion underwent only gastroscopy (174, 33.5%) and even fewer underwent only colonoscopy (60, 11.5%). Intraoperative hypotension occurred in 201 patients (38.7%), while intraoperative hypoxemia occurred in 166 (31.9%). Endotracheal intubation was not required during the study.

### 3.1 Identification of risk factors for SRAEs

Patients were randomly assigned in a 7:3 ratio to either the training set (*n* = 364) or the validation set (*n* = 156), and the two sets did not differ significantly in any of the baseline clinicodemographic characteristics that we examined (Table 1). In contrast, several baseline variables differed significantly between patients who experienced at least one SRAE or not within the training and validation sets (Table 2). The variables that differed significantly in the training set were coded (Table 3) and entered into Firth-penalized multivariable logistic regression which identified the following independent risk factors for SRAEs (Table 4): advanced age, history of snoring, frailty, preoperative hypertension, chronic obstructive pulmonary disease, prolonged fasting before the procedure, and increased initial dose of etomidate-propofol. Conversely, regular physical activity was found to be a protective factor.

**TABLE 1:** Baseline characteristics of patients in the training and validation sets.

Characteristic	Training set (*n* = 364)	Validation set (*n* = 156)	*t*/χ^2^/*z*	*P*
Sex			0.529	0.467
Men	190 (52.2)	76 (48.7)		
Women	174 (47.8)	80 (51.3)		
Age, yr	68 ± 7	68 ± 6	0.734	0.511
Body mass index, kg/m^2^	23.2 ± 2.8	23.7 ± 3.5	1.572	0.117
Status on the American Society of Anesthesiologists scale			1.467	0.690
I	25 (6.9)	9 (5.8)		
II	250 (68.7)	102 (65.4)		
III	89 (24.5)	45 (28.9)		
Smoking	54 (14.8)	16 (10.3)	1.965	0.161
Alcohol consumption	94 (25.8)	41 (26.3)	0.012	0.913
Regular physical activity	262 (72.0)	106 (68.0)	0.857	0.355
History of snoring			6.449	0.092
None	204 (56.0)	95 (60.9)		
Mild (grade 1)	104 (28.6)	29 (18.6)		
Moderate (grade 2)	38 (10.4)	21 (13.5)		
Severe (grade 3)	18 (5.0)	11 (7.1)		
Frailty			2.361	0.307
Robust	131 (36.0)	48 (30.8)		
Pre-frail	182 (50.0)	79 (50.6)		
Frail	51 (14.0)	29 (18.6)		
**Comorbidities**				
Hypertension	119 (32.7)	58 (37.2)	0.979	0.322
Coronary artery disease	57 (15.7)	24 (15.4)	0.006	0.937
COPD	152 (41.8)	59 (37.8)	0.702	0.402
Renal insufficiency	34 (9.3)	15 (9.6)	0.010	0.922
Diabetes mellitus	55 (15.1)	20 (12.8)	0.464	0.496
Fasting time before procedure, h	17(14.0,18.0)	17(16.0,18.0)	1.430	0.153
Endoscopy type			1.671	0.434
Gastroscopy	121 (33.2)	53 (34.0)		
Colonoscopy	38 (10.4)	22 (14.1)		
Both	205 (56.3)	81 (51.9)		
Sufentanil dosage, μg	2.3 ± 0.7	2.2 ± 0.6	0.482	0.630
Initial EP mixture, mL	12 ± 1.9	11.9 ± 2.1	0.476	0.634
**Change from baseline (%) in**				
Systolic blood pressure	17.0 (11.0, 23.0)	18.0 (12.0, 22.0)	−1.274	0.203
Diastolic blood pressure	18.0 (10.0, 25.0)	18(11.0, 24.0)	−0.860	0.390
Heart rate	0.0 (13.0)	0.0 (9.2)	−0.317	0.752
Hypotension	142 (39.0)	59 (37.8)	0.065	0.798
Hypoxemia	122 (33.5)	44 (28.2)	1.418	0.234
Body movement	21 (5.8)	11 (7.1)	0.311	0.577
Coughing	11 (3.0)	4 (2.6)	0.082	0.775

The values are descriptive statistics for the entire training and validation cohorts. Values are n (%), mean ± SD, or median (interquartile range), unless otherwise noted. COPD, chronic obstructive pulmonary disease; EP, etomidate-propofol.

**TABLE 2:** Comparison of baseline characteristics and perioperative variables between patients who experienced at least one SRAE or not in the training and validation sets.

Characteristic	Training set		*P*	Validation set		*P*
	No SRAE (*n* = 198)	SRAE (*n* = 166)		No SRAE *n* = 95	SRAE *n* = 61	
Sex			0.548			0.701
Men	100(50.5)	90(54.2)		44(46.3)	31(50.8)	
Women	98(49.5)	76(45.8)		51(53.7)	30(49.2)	
Age, yr	67.9 ± 5.6	69.3 ± 7.2	0.033	65.8 ± 5.9	68.7 ± 6.6	0.006
Body mass index, kg/m^2^	22.8 ± 2.8	23.5 ± 2.6	0.014	22.8 ± 3.8	23.8 ± 3.3	0.084
Status on the American Society of Anesthesiologists scale			0.014			0.012
I	12(6.0)	13(7.8)		3(3.2)	6(9.8)	
II	116(58.6)	134(80.7)		57(60.0)	45(73.8)	
III	70(35.3)	19(11.4)		35(36.8)	10(16.4)	
Smoking	2(1.0)	52(30.7)	< 0.001	2(2.1)	14(23.0)	< 0.001
Alcohol consumption	38(19.2)	56(33.7)	0.002	38(40.0)	5(8.2)	< 0.001
Regular physical activity	189(95.5)	73(44.0)	< 0.001	87(91.6)	19(31.1)	< 0.001
History of snoring			< 0.001			<0.001
None	133(67.2)	64(38.6)		71(74.7)	28(45.9)	
Mild (grade 1)	63(31.8)	49(29.5)		24(25.3)	15(24.6)	
Moderate (grade 2)	2(1.0)	35(21.1)		0(0.0)	11(18.0)	
Severe (grade 3)	0(0.00)	18(10.9)		0(0.0)	7(11.5)	
Frailty			< 0.001			<0.001
Robust (0 points)	81(41.0)	51(30.7)		40(42.1)	19(31.2)	
Pre-frail	117(59.1)	64(38.6)		52(54.7)	16(26.2)	
Frail	0(0.00%)	51(30.7)		3(3.2)	26(42.6)	
**Comorbidities**						
Hypertension	54(27.3)	65(38.6)	0.029	36(37.9)	18(29.5)	0.367
Coronary artery disease	16(9.1)	36(23.5)	< 0.001	17(17.9)	7(11.5)	0.608
COPD	30(15.2)	122(73.5)	<0.001	15(15.8)	44(72.1)	< 0.001
Renal insufficiency	21(12.1)	12(7.8)	0.241	9(11.6)	6(11.5)	0.454
Diabetes mellitus	30(15.2)	25(15.7)	0.543	10(10.5)	10(16.4)	0.409
Fasting time before procedure, h	17(16.0,18.0)	17(15.0,19.0)	0.02	17(16.0,19.0)	17(13.0,18.0)	0.01
Endoscopy type			0.514			0.92
Gastroscopy	66(38.4)	53(25.9)	34(35.8)	20(32.8)		
Colonoscopy	20(10.1)	17(10.2)	13(13.7)	9(14.8)		
Both	112(51.5)	96(63.8)	48(50.5)	32(52.5)		
Sufentanil dosage, μg	2.2 ± 0.5	2.4 ± 0.7	0.001	2.1 ± 0.5	2.3 ± 0.7	0.125
Initial EP mixture, mL	12.0 ± 1.5	12.3 ± 1.9	0.037	11.9 ± 1.3	12.4 ± 1.8	0.081
**Change from baseline in (%)**						
Systolic blood pressure	17 (10.5, 23.5)	17 (11.0, 23.0)	0.534	18 (14.0,23.0)	17 (13.0,22.0)	0.513
Diastolic blood pressure	17 (10.0,24.0)	19 (11.0, 26.0)	0.185	18 (12.0, 24.0)	18 (12.0,26.0)	0.893
Heart rate	1 (−9.0,5.0)	−1.0 (−9.0, 5.0)	0.093	0 (−5.0,3.0)	0(−5.0,7.0)	0.437

ASA, American Society of Anesthesiologists; COPD, chronic obstructive pulmonary disease; EP, etomidate-propofol; SRAE, sedation-related adverse event.

**TABLE 3:** Definitions of variables in logistic regression.

Variable	Name	Description
Sex	X1	Male = 1, Female = 2
Age, yr	X2	60–69 = 1, 70–79 = 2, 80 + = 3
Body mass index, kg/m^2^	X3	<24 = 1, 24–28 = 2, > 28 = 3
ASA classification	X4	I-II = 1, III = 2
Smoking	X5	No = 0, Yes = 1
Alcohol consumption	X6	No = 0, Yes = 1
Regular physical activity	X7	No exercise = 0, Regular exercise = 1
History of snoring	X8	None = 0, Mild = 1, Moderate = 2, Severe = 3
Frailty status	X9	Robust = 0, Pre-frail = 1, Frail = 2
Hypertension	X10	None = 0, Stage 1 = 1, Stage 2 = 2
Coronary artery disease	X11	No = 0, Yes = 1
COPD	X12	No = 0, Yes = 1
Renal insufficiency	X13	No = 0, Yes = 1
Diabetes mellitus	X14	No = 0, Yes = 1
Fasting time before procedure	X15	≤ 20 h = 0, > 20 h = 1
Endoscopy type	X16	Gastroscopy = 0, Colonoscopy = 1, Both = 2
Sufentanil dosage, μg	X17	1–3 μg = 0, 4–5 μg = 1
Initial EP dose, mL	X18	≤8 mL = 0, 9–15 mL = 1, > 15 mL = 2

ASA, American Society of Anesthesiologists; COPD, chronic obstructive pulmonary disease; EP, etomidate-propofol.

**TABLE 4:** Firth-penalized multivariable logistic regression to identify risk factors of sedation-related adverse events in the training set.

Variable	B	Wald	*P*	Exp (B)	95% confidence interval
Age	1.56	9.52	0.01	4.76	1.66–8.66
Regular physical activity	−2.35	5.02	0.02	0.09	0.04–0.38
History of snoring	1.76	12.40	0.001	5.83	1.80–7.29
Frailty	1.92	4.28	0.03	6.84	2.33–9.50
Hypertension	1.32	5.76	0.02	3.74	1.81–6.10
Chronic obstructive pulmonary disease	0.80	4.98	0.02	2.23	1.33–7.21
Fasting time before procedure	1.40	6.32	0.003	4.04	3.31–10.32
Initial etomidate-propofol dose	2.21	8.85	0.007	9.14	3.94–13.73
Constant	−5.732	23.72	0.001	0.001	Not applicable

OR and 95% CI were estimated using Firth’s penalized-likelihood logistic regression to reduce small-sample bias and potential quasi-complete separation.

### 3.2 Development and internal validation of a nomogram to predict SRAEs

**FIGURE 2:**
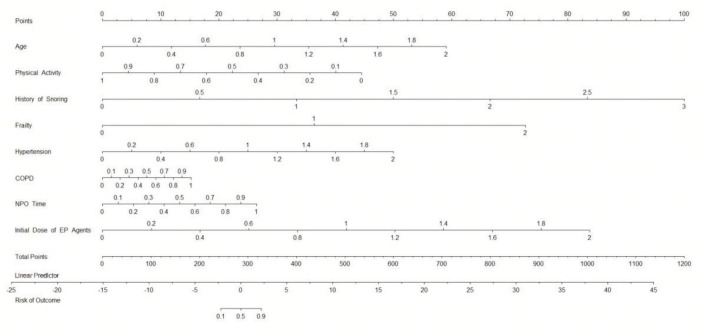
Nomogram to predict sedation-related adverse events in elderly patients undergoing painless gastrointestinal endoscopy. The nomogram incorporates eight independent risk factors identified through multivariable logistic regression of data in the training set. COPD, chronic obstructive pulmonary disease.

**FIGURE 3:**
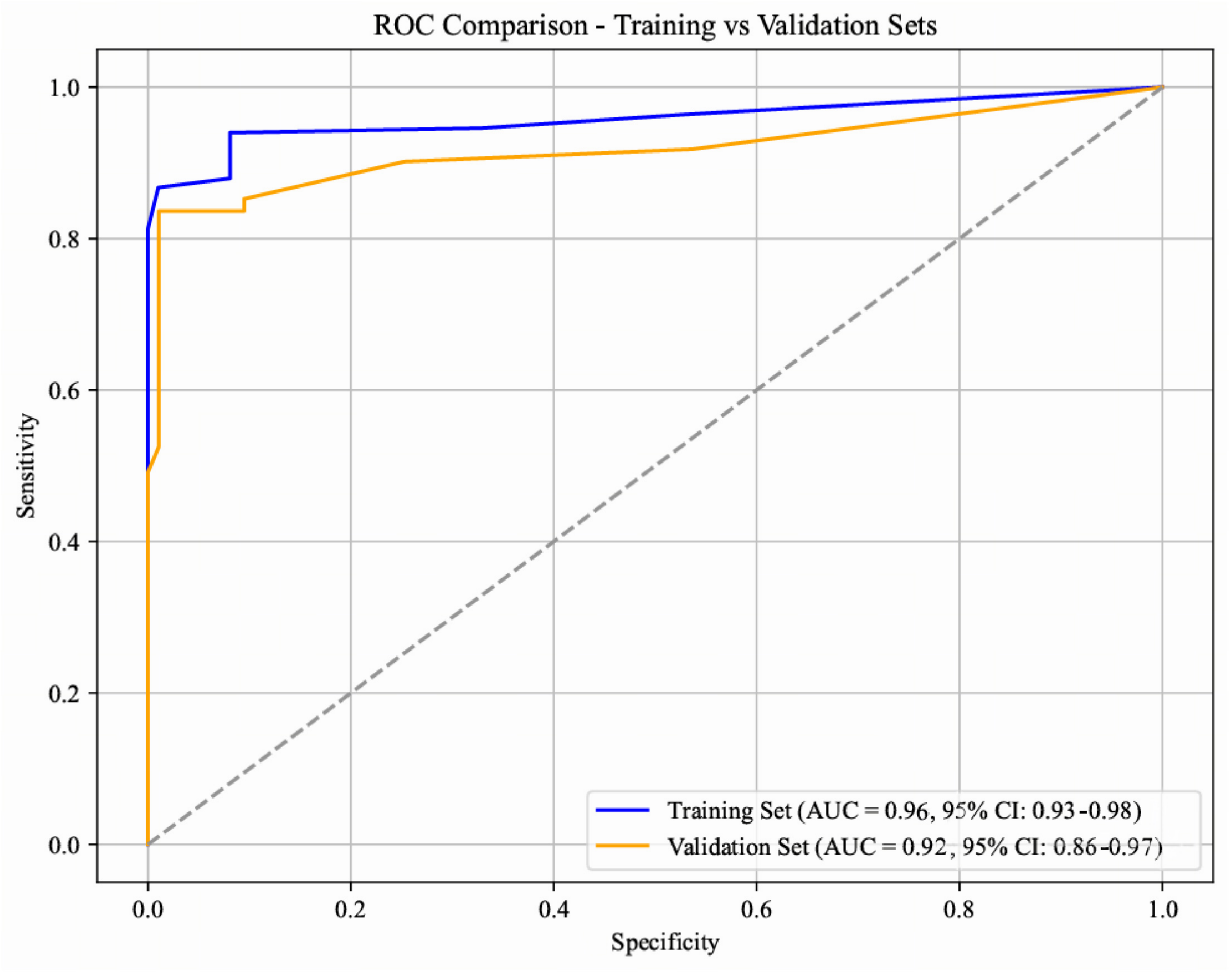
Receiver operating characteristic curves to assess the ability of the nomogram to predict sedation-related adverse events in the training set (blue) or validation set (orange). The areas under the curves and corresponding 95% confidence intervals are shown at the lower right. The diagonal dotted line represents a non-discriminative model (AUC = 0.5).

A nomogram was constructed based on the eight independent risk factors of SRAEs identified through Firth-penalized multivariable logistic regression (Figure 2). ROC analysis demonstrated excellent discriminative ability of the Firth-penalized logistic regression model (Figure 3). In the training set, the area under the ROC curve (AUC) was 0.96 (95% CI, 0.93–0.98). In the independent validation set, the AUC remained high at 0.92 (95% CI, 0.86–0.97), indicating only a modest decrease in performance. These findings suggest that the nomogram provides strong and stable discrimination between patients with and without SRAEs in both the development and validation cohorts.

**FIGURE 4:**
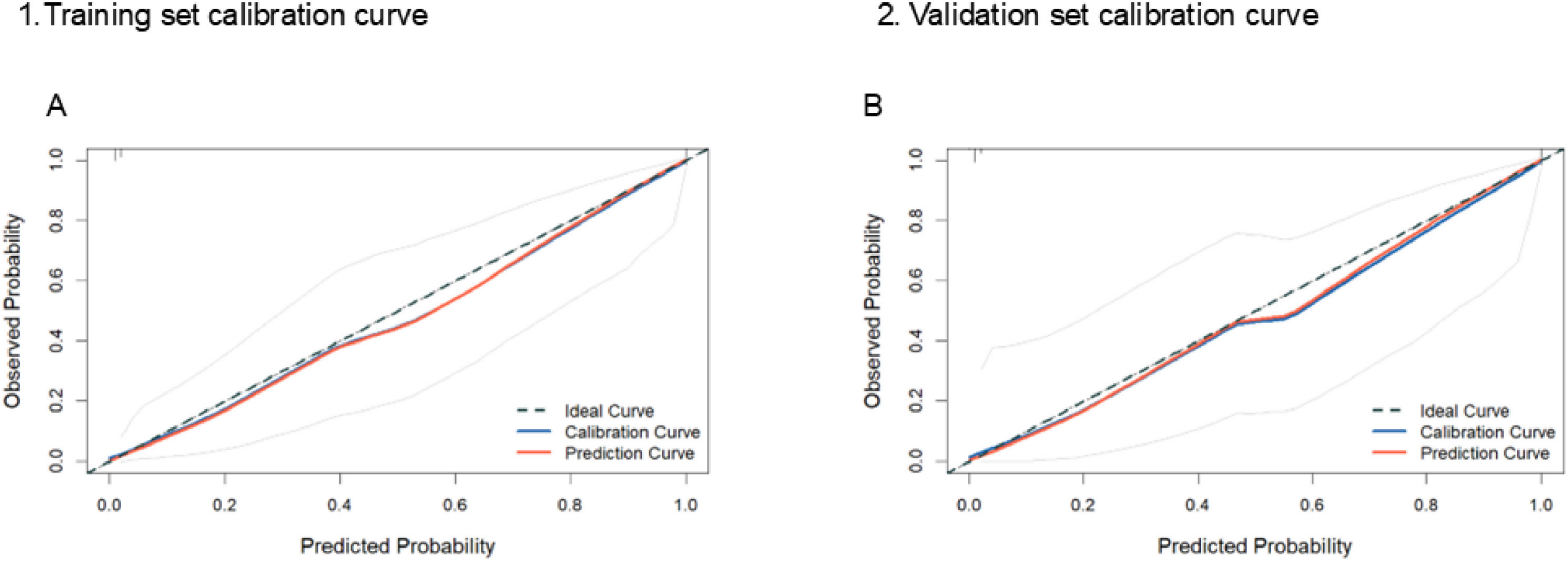
Calibration curves of the nomogram when applied to the **(A)** training set or **(B)** validation set. The diagonal dotted line represents perfect prediction.

Next, calibration plots showed good agreement between the predicted and observed probabilities of SRAEs in both the training and validation sets (Figure 4). In the training set, the bootstrapped calibration curve nearly overlapped the 45° ideal line across the full range of predicted risks, indicating minimal optimism and excellent calibration. In the validation set, the calibration curve also closely followed the ideal line, with only slight deviations at intermediate predicted probabilities, suggesting that the Firth-penalized logistic regression model provides reliable absolute risk estimates in an independent cohort. The Hosmer–Lemeshow test showed *P* = 0.41 in the training set and *P* = 0.63 in the validation set, indicating good agreement between the predicted and observed probabilities.

### 3.3 Clinical utility of the nomogram

**FIGURE 5:**
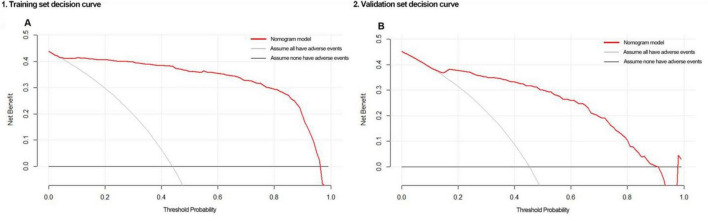
Decision curves of the nomogram when applied to the **(A)** training set or **(B)** validation set.

Decision curves indicated that the nomogram showed good clinical utility across a broad range of threshold probabilities, whether in the training set (5–96%) or validation set (7–93%) (Figure 5).

## 4 Discussion

In this study, perioperative data from 520 elderly patients undergoing painless gastrointestinal endoscopy were used to develop a nomogram to predict SRAEs based on eight independent risk factors, which performed well in internal validation and showed clinical utility across a broad range of threshold probabilities. This model may help identify individuals at high risk of SRAEs and personalize perioperative procedures. Because the nomogram incorporates the initial etomidate–propofol dose, it is best interpreted as an intra-procedural risk to assess the ability ment tool rather than a purely pre-procedural screening model. In practice, this model can help inform the choice of sedation strategy, further refine risk stratification, and guide subsequent intra-procedural management.

Anesthesia in our study was induced with intravenous sufentanil citrate 0.05 μg/kg and lidocaine 0.3 mg/kg, followed by a slow bolus of a 1:1 etomidate–propofol mixture (0.2 mL/kg) and top-up doses of 0.07 mL/kg every 5 min as needed. This regimen corresponds to deep sedation without tracheal intubation and is widely used in China because etomidate provides relatively stable hemodynamics (15), whereas propofol increases risk of hypotension and respiratory depression (16, 17) but offers rapid onset and recovery. However, our observed incidences of intraoperative hypotension (38.7%) and hypoxemia (31.9%) indicate that this protocol is not risk-free in elderly patients with multiple comorbidities. Despite standardized treatment of hypotension with intravenous ephedrine (3–6 mg) and of sinus bradycardia with atropine (0.3–0.5 mg), clinically relevant hemodynamic and respiratory events remained common in this vulnerable population. Compared with previous studies of propofol-based deep sedation in mixed-age adults undergoing gastrointestinal endoscopy, where hypotension and hypoxemia are typically reported in up to approximately 36 and 20% of patients (2, 3), respectively, our rates are at the upper end of these ranges. This likely reflects the higher baseline risk in our cohort, which consisted exclusively of patients ≥ 60 years with a substantial burden of hypertension and chronic obstructive pulmonary disease and relatively long fasting times. Importantly, we identified a higher initial etomidate–propofol dose as an independent predictor of SRAEs, highlighting that the choice and dosing of sedative agents are modifiable risk factors. These findings suggest that, in elderly patients, more conservative dosing strategies and careful titration of etomidate–propofol, or the use of alternative sedative regimens, should be considered to reduce the risk of hypotension and hypoxemia.

The independent predictors of SRAEs in our sample are consistent with age-related decline. As individuals grow older, arteriosclerosis reduces vascular elasticity and the autonomic regulatory system becomes weaker, increasing risk of intraoperative hypotension. Frailty is associated with lower physiological reserves across multiple systems, rendering patients more vulnerable to the hemodynamic stress of anesthesia induction (18, 19). For example, frailty may reduce oxygenation (20), increasing the risk of intraoperative hypoxemia. Indeed, hypotension and hypoxemia were more frequent among our frail patients than among non-frail patients, raising the possibility that conventional anesthesia protocols may fail to meet the perfusion and oxygenation demands of this population. Future research should explore this in detail.

Longer fasting time before endoscopy was associated with higher risk of SRAEs in our sample. Elderly patients may be more sensitive to fluid imbalance than younger individuals (21), and the hypovolemia arising from extended fasting may exacerbate the already strong variations in blood pressure and heart rate that older patients experience during gastroscopy (22). It may be necessary to tailor fluid management strategies for elderly undergoing gastroscopy, which should be explored in future work.

History of snoring predicted intraoperative hypoxemia in our sample, consistent with the known association between such a history and risk of airway obstruction during anesthesia due to mandibular relaxation, posterior tongue displacement or upper airway collapse (23, 24). Chronic obstructive pulmonary disease also predicted intraoperative hypoxemia in our sample, probably reflecting that compromised alveolar function, ventilation–perfusion mismatch, chronic inflammation, and airway hyperresponsiveness in COPD can further impair oxygenation during anesthesia (25).

Regular physical activity protected against SRAEs in our sample. Moderate exercise is known to enhance cardiopulmonary function, improve oxygen uptake and use, delay age-related organ decline, and promote metabolic efficiency. Exercise-based interventions can improve postoperative outcomes and reduce risk of complications in elderly with gastrointestinal malignancies (26). Our study extends these findings to the context of sedation for painless endoscopy. Exercise and potentially other lifestyle factors may be important to consider when stratifying patients by SRAE risk and when planning their sedation before the procedure and their management afterward.

Our study substantially extends the literature on SRAEs by focusing on elderly and defining a composite endpoint of either hypotension or hypoxemia, rather than only one SRAE (27). It considered factors related to lifestyle (e.g., physical activity, snoring), physiological status (frailty), and the perioperative experience (preoperative fasting time, initial dose of etomidate-propofol) when predicting SRAE risk. Our finding that fasting time before the procedure appeared in the final nomogram underscores the importance of proper preparation of elderly patients before the procedure.

At the same time, our findings should be interpreted with caution because they are derived from a relatively small, single-center cohort. All patients were elderly Chinese individuals treated at Wenjiang District People’s Hospital of Chengdu, an urban–suburban setting with a relatively high prevalence of hypertension and chronic obstructive pulmonary disease, which may limit the generalizability of our results to other regions and ethnicities. The incidences of intraoperative hypotension (38.7%) and hypoxemia (31.9%) in this cohort are at the upper end of the ranges reported in mixed-age populations undergoing propofol-based sedation for gastrointestinal endoscopy [hypotension up to ∼36% and hypoxemia around 20% (2, 3)]. This likely reflects the inclusion of only patients ≥ 60 years, the higher burden of cardiovascular and respiratory comorbidities, and relatively long fasting times (median 17 h). Thus, although the identified predictors of SRAEs—such as age, snoring, frailty, hypertension, COPD, and prolonged fasting—are biologically plausible and probably relevant across settings, the absolute risk estimates from our nomogram should be externally validated and, if necessary, recalibrated in other hospitals, regions, and ethnic groups before routine clinical use. In addition, although our model showed excellent internal discrimination, the very high AUC values and large odds ratios for some predictors raise concerns about overfitting or quasi-complete separation. We therefore refitted the model using Firth’s penalized-likelihood logistic regression, which produced attenuated but still significant effects for key predictors and more conservative estimates, although residual overfitting cannot be excluded. Furthermore, we categorized several continuous predictors such as age and BMI using fixed cutoffs to improve clinical interpretability and to maintain model stability with a relatively modest number of events. This approach may have led to some loss of information and reduced statistical power, and future multicenter studies should consider modeling these predictors as continuous variables using flexible, data-driven approaches (e.g., splines) to better capture potential non-linear relationships.

## Data Availability

The raw data supporting the conclusions of this article will be made available by the authors, without undue reservation.
