# Supplementary material for: A nomogram for predicting sedation-related adverse events in elderly patients undergoing painless gastrointestinal endoscopy
Source: Front Med (Lausanne). 2026 Jan 12;12:1713720. doi: 10.3389/fmed.2025.1713720 (PMC12832725; doi:10.3389/fmed.2025.1713720)
Supplement: Supplementary file 1 [file Table_1.docx]

# CONSORT Checklist

Manuscript title: A nomogram for predicting sedation-related adverse events in elderly patients undergoing painless gastrointestinal endoscopy

Note: This study is a prospective observational trial rather than a randomized controlled trial. Therefore, items specifically related to randomization, allocation concealment, and blinding are marked as 'Not applicable'.

## Title and Abstract

1a: Not applicable (observational study, not a randomised trial).

1b: Structured abstract provided, including background, objective, methods, results, conclusion, and trial registration.

## Introduction

2a: Scientific background and rationale described in Introduction.

2b: Objective clearly stated: to develop and validate a predictive model (nomogram) for SRAEs in elderly undergoing painless endoscopy.

## Methods

3a: Not applicable (no randomisation, observational design).

3b: No important changes to methods after trial commencement.

4a: Eligibility criteria described in Methods, Section 2.1.

4b: Single-centre study at Wenjiang District People's Hospital, Chengdu, China.

5: Sedation and management protocols described in detail (Section 2.3).

6a: Primary outcome: sedation-related adverse events (hypotension or hypoxemia).

6b: No changes to outcomes after commencement.

7a: Sample size rationale provided (≥20 patients per variable, Section 2.2).

7b: Not applicable (no interim analyses or stopping guidelines).

8a: Not applicable (no randomisation).

8b: Not applicable.

9: Not applicable.

10: Not applicable.

11a: Not applicable (observational design, no blinding).

11b: Not applicable.

12a: Statistical methods described: logistic regression, ROC curves, calibration, decision curve analysis.

12b: Additional analyses reported: subgroup comparisons, bootstrap validation.

## Results

13a: Not applicable (no randomisation). Flow diagram provided (Figure 1).

13b: Exclusions reported (incomplete data, lost to follow-up).

14a: Recruitment and follow-up between April 2023 and June 2024.

14b: Not applicable (study completed as planned).

15: Baseline demographics and clinical characteristics in Table 1.

16: Analysis sets described: training (n=364) and validation (n=156).

17a: Results reported with effect estimates: OR, CI, AUC values.

17b: Not applicable (binary outcomes reported as ORs, not relative risks).

18: Additional analyses included calibration plots, decision curve analysis.

19: Intraoperative adverse events (hypotension, hypoxemia) reported.

## Discussion

20: Limitations discussed: single-centre, relatively small sample, no external validation.

21: Generalisability addressed (elderly Chinese patients, external validation needed).

22: Interpretation consistent with results, balanced against literature.

## Other information

23: Trial registration: Chinese Clinical Trial Registry (ChiCTR2300069816), registered March 27, 2023.

24: Protocol available upon request from corresponding author.

25: Funding sources disclosed in Declarations (Sichuan Province, Chengdu Health Commission, Sichuan Medical Association).
